# The Effects of Verapamil, Hydralazine, and Doxazosin on Renin, Aldosterone, and the Ratio Thereof

**DOI:** 10.1007/s10557-021-07262-3

**Published:** 2021-09-13

**Authors:** Gregory P. Veldhuizen, Rawan M. Alnazer, Peter W. de Leeuw, Abraham A. Kroon

**Affiliations:** Dept of Internal Medicine, Maastricht University Medical Center and Cardiovascular Research Institute Maastricht (CARIM), PO Box 5800, Maastricht, 6202 AZ The Netherlands

**Keywords:** Renin-aldosterone ratio, Verapamil, Hydralazine, Doxazosin

## Abstract

**Purpose:**

Hydralazine, doxazosin, and verapamil are currently recommended by the Endocrine Society as acceptable bridging treatment in those in whom full cessation of antihypertensive medication is infeasible during screening for primary aldosteronism (PA). This is under the assumption that they cause minimal to no effect on the aldosterone-to-renin ratio, the most widely used screening test for PA. However, limited evidence is available regarding the effects of these particular drugs on said ratio.

**Methods:**

In the present study, we retrospectively assessed the changes in aldosterone, renin, and aldosterone-to-renin values in essential hypertensive participants before and after treatment with either hydralazine (*n* = 26) or doxazosin (*n* = 20) or verapamil (*n* = 15). All samples were taken under highly standardized conditions.

**Results:**

Hydralazine resulted in a borderline significant rise in active plasma renin concentration (19 vs 25 mIU/L, *p* = 0.067) and a significant fall in the aldosterone-to-renin ratio (38 vs 24, *p* = 0.017). Doxazosin caused declines in both plasma aldosterone concentration (470 vs 330 pmol/L, *p* = 0.028) and the aldosterone-to-renin ratio (30 vs 20, *p* = 0.020). With respect to verapamil, we found no statistically significant effect on any of these outcome variables.

**Conclusion:**

We conclude that the assumption that these drugs can be used with little consequence to the aldosterone-to-renin cannot be substantiated. While it is possible that they are indeed the best option when full antihypertensive drug cessation is infeasible, the potential effects of these drugs must still be taken into account when interpreting the aldosterone-to-renin ratio.

## Introduction

The aldosterone-to-renin ratio (ARR) is the most widely used screening test for primary aldosteronism (PA) and is recommended by current guidelines [1]. However, due to the potential effects that antihypertensive drugs may have on the renin–angiotensin–aldosterone system (RAAS), these guidelines advocate to cease antihypertensive medication prior to ARR screening whenever possible. In many patients, however, such a strategy is infeasible. In the event that cessation of antihypertensive agents is not possible, the current Endocrine Society guidelines recommend switching to verapamil, hydralazine, or an α-adrenergic blocker such as doxazosin on the assumption that these drugs exert no-to-minimal effects on the RAAS [1]. While the theoretical background for their largely neutral effect on the ARR is strong, a recent review article found that no robust studies investigating the effects of verapamil and hydralazine on the ARR have been performed [2]. Specifically, studies have usually assessed average changes in renin and aldosterone but not the individual changes in ARR. In addition, only one study has addressed the effects of doxazosin and very notably showed that both aldosterone and ARR values significantly fell during treatment with that drug [3]. The objective of the present study, therefore, is to investigate the effects that verapamil, hydralazine, and doxazosin may have on the ARR in individual patients. To this end, we reassessed the data from three patient groups which we studied earlier for changes in renin and aldosterone during those treatments. We hypothesized that none of the medications under scrutiny would significantly affect the ARR.

## Patients and Methods

### Study Population

A total of 61 Caucasian Dutch patients who had been referred to our hypertension clinic for evaluation of their hypertension participated in the three studies. Of these, 26, 20, and 15 had been treated with hydralazine, doxazosin, and verapamil, respectively. Except for being hypertensive, none of the patients had any concomitant disease, such as diabetes or reduced renal function. Nevertheless, slightly over half of them were overweight.

All active treatment phases were preceded by a placebo run-in period.

All participants gave informed consent to partake in this study which was approved by the Institutional Review Board.

Secondary causes of hypertension were ruled out after appropriate diagnostic procedures. These included measurements of active plasma renin concentration (APRC), plasma aldosterone concentration (PAC), calculation of the ARR, and additional tests such as saline loading if indicated. In case secondary hypertension was diagnosed, patients were excluded from this study.

### Procedures

As part of our routine diagnostic work-up for secondary hypertension, we discontinued any antihypertensive medication for 3 weeks when this could be safely withheld. All measurements took place within this time window. After the initial assessment, during which participants were given a placebo tablet, they were treated with the respective drugs for at least 2 weeks prior to repeat testing. Doses were up-titrated until blood pressure was 140/90 mmHg or below.

All investigations took place in a metabolic ward to which patients had been admitted prior to the investigations. On the day of testing, after an overnight fast and complete bed rest for 10 h, participants stayed in the laboratory from 08:00 a.m. until noon. After we had inserted an indwelling needle into the right antecubital vein, patients were allowed to rest for another 45 min. Thereafter, we took the first blood sample for determination of untreated plasma active renin and untreated plasma aldosterone concentrations (U-APRC and U-PAC respectively). All blood samples were taken in the supine position. Blood was collected in chilled tubes and spun immediately under cooled conditions and the plasma was stored at − 80 °C until assay. This process was repeated after participants had been placed on their respective drug regimens to determine their treated APRC and PAC values (T-APRC and T-PAC respectively).

Throughout the entire period, blood pressure was measured at 5-min intervals with an automatic, oscillometric device (Dinamap, Tampa, FL), while patients remained in the supine position until the end of the study.

APRC was measured by a direct immunoradiometric assay that detects active renin. Its characteristics are as follows: sensitivity 2.5 mIU/L, intra-assay variability 2.6%, and inter-assay variability 4.3%. PAC was measured by solid-phase radioimmunoassay (antibody-coated tubes) with a sensitivity of 55 pmol/L, an intra-assay variability of 4.3%, and an inter-assay variability of 6.7%. Untreated and treated ARR (U-ARR and T-ARR, respectively) were calculated from the respective PAC and APRC results.

### Statistical Methods

Using IBM SPSS Statistics Version 26 for Windows for all analyses, we first confirmed the normality of distribution of all data. The ones from the verapamil and doxazosin populations (VP and DP respectively) were both normally distributed, but those from the hydralazine population (HP) required log transformations before analysis. We performed paired t-tests on all groups comparing U-APRC and T-APRC, U-PAC and T-PAC, and U-ARR and T-ARR. Multiple regression analyses were performed to assess possible confounding by age, sex, and BMI on changes in APRC, PAC, and ARR during treatment.

For all analyses, a *p* value of < 0.05 was considered statistically significant.

Unless stated otherwise, data are expressed as means with standard error of the mean (SEM).

## Results

The demographic characteristics of all participants are summarized in Table 1. Changes in renin, aldosterone, and the ARR during treatment are displayed in Table 2.

**Table 1:** Demographic baseline characteristics of all study populations

	Hydralazine (*n* = 26	Doxazosin (*n* = 21)	Verapamil (*n* = 15
Male:female (ratio)	7:19	14:6	9:6
Age (years)	46 (3)	50 (2)	45 (3)
Height (males) (cm)	177 (2)	176 (2)	177 (2)
Height (females) (cm)	163 (1)	165 (1)	164 (1)
Weight (males) (kg)	85 (2)	83 (2)	84 (4)
Weight (females) (kg)	67 (3)	71 (3)	70 (4)
BMI (kg/m^2^)	25 (1)	27 (1)	27 (1)
Participants with BMI > 25 (%)	54%	55%	60%
Systolic blood pressure (mmHg)	159 (5)	165 (4)	152 (6)
Diastolic blood pressure (mmHg)	98 (3)	102 (3)	95 (4)
Mean arterial pressure	119 (3)	124 (3)	113 (4)
Heart rate (BPM)	72 (2)	76 (3)	69 (2)
Plasma sodium (mmol/L)	143 (1)	141 (1)	140 (1)
Plasma potassium (mmol/L)	4.2 (0.2)	4.4 (0.1)	4.3 (0.1)
Plasma creatinine (µmol/L)	99 (9)	104 (6)	90 (5)

All data expressed as means and standard error of the mean (SEM)

**Table 2:** Effect of the medications on average levels of PAC, APRC, and ARR

		Untreated	Treated	*p* value
Hydralazine	PAC	410 (68)	360 (58)	0.216
	APRC	19 (3)	25 (4)	0.067
	ARR	38 (10)	24 (4)	0.017
Doxazosin	PAC	470 (49)	330 (28)	0.028
	APRC	19 (2)	20 (2)	0.639
	ARR	30 (5)	20 (3)	0.020
Verapamil	PAC	420 (58)	326 (35)	0.141
	APRC	21 (3)	21 (4)	0.840
	ARR	27 (8)	18 (3)	0.188

All data expressed as means and standard error of the mean (SEM)

### Hydralazine

Hydralazine was administered at a median dosage of 150 mg per day (interquartile range (IQR) 75–150 mg). During treatment with hydralazine, APRC rose from 19 ± 3 to 25 ± 4 mIU/L, which was of borderline statistical significance (*p* = 0.067). PAC, on the other hand, did not change significantly (410 ± 68 vs 360 ± 58 pmol/L, *p* = 0.216). The ARR fell significantly from 38 ± 10 to 24 ± 4 (*p* = 0.017). Multiple regression analysis did not reveal age, sex, BMI, baseline blood pressure, or drug dosage to be significant predictors for the percentage change in APRC, PAC, and ARR values.

### Doxazosin

Doxazosin was administered at a median dosage of 10 mg per day (IQR 3–20 mg). Doxazosin did not significantly affect APRC levels post-treatment (19 ± 2 vs 20 ± 2 mIU/L, *p* = 0.639). However, declines in PAC (470 ± 49 vs 330 ± 28 pmol/L) and ARR (30 ± 5 vs 20 ± 3) were both statistically significant (*p* = 0.028 and *p* = 0.020, respectively). Multiple regression analysis did not reveal age, sex, BMI, baseline blood pressure, or drug dosage to be significant predictors for the percentage change in APRC, PAC, and ARR values.

### Verapamil

Verapamil was administered at a median dosage of 240 mg per day (IQR 240–480 mg). The mean values for pre- and post-treatment levels of APRC (21 ± 3 vs 21 ± 4 mlU/L) did not significantly differ. PAC (420 ± 58 vs 326 ± 35 pmol/L) and the ARR (27 ± 8 vs 18 ± 3) fell after administration of verapamil, but changes were very variable and not statistically significant (*p* = 0.141 and *p* = 0.188 respectively).

**Fig. 1:**
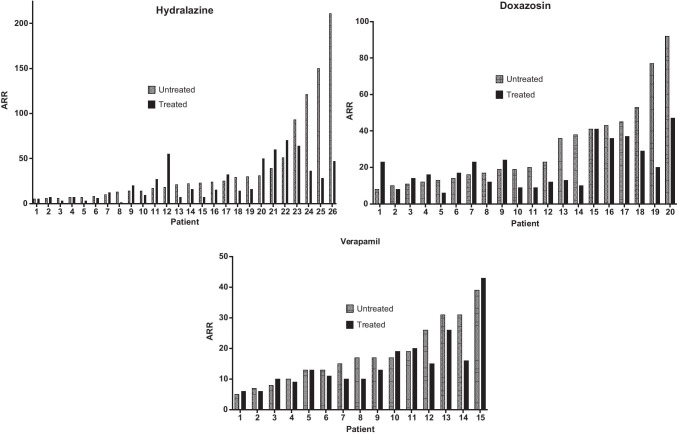
Aldosterone-to-renin ratio (ARR) of individual participants comparing untreated and treated values, arranging in order of ascending untreated ARR values

Individual patient data of the ARR before and after the three forms of treatment are presented in Fig. 1.

## Discussion

The primary findings of our study are that both hydralazine and doxazosin resulted in significant falls in the ARR, an outcome not in keeping with conventional wisdom on the topic. Hydralazine’s changes seem to have been primarily driven by changes in the APRC while doxazosin’s effect seemed to have conversely been the result of changes in PAC. In addition, while the changes in PAC and ARR values after verapamil administration were not statistically significant, the magnitude of their declines was nonetheless rather large in our opinion. Given that verapamil was our smallest study population, comprising only 15 participants, it is possible that a larger population would garner a clearer picture regarding verapamil’s potential effects on the RAAS. Regardless, we feel that it would be negligent to conclude that verapamil has no effect on the ARR based on the non-significant *p* value obtained in our statistical analysis.

The effect of hydralazine on aldosterone levels has been largely overlooked in scientific research, with no studies regarding its effect on the ARR published. A number of studies could be found regarding the effect of hydralazine on renin levels. In all studies, hydralazine administration resulted in rises in renin levels, with the rise being statistically significant in all but one study [4–9]. Hydralazine has been found in previous studies to result in increased sympathetic outflow [10–13] and, hence, increased renin release [14–17]. This interplay between increased sympathetic outflow and β_1_-adrenoceptor-mediated renin activation could be the driving factor in our findings regarding hydralazine.

The most notable study concerning the effect of doxazosin on aldosterone, renin, and the ARR was performed by Mulatero et al. in a population with primary aldosteronism [3]. The findings of that study are well in line with our own, with significant declines in PAC and ARR being noted, as well as a statistically insignificant effect on APRC. The magnitude of these effects was considerably smaller than in our study. However, doxazosin’s apparent aldosterone-suppressing effects would be presumably dampened by the autonomous excessive production of aldosterone that typifies PA. Thus, the study by Mulatero et al. and our own could dovetail nicely in demonstrating the spectrum of the effects that doxazosin has on the two primary patient types who typically undergo PA screening, that being essential hypertensives and patients with as-yet undiagnosed PA. Of the few other studies performed regarding doxazosin, they typically focused on the effect it had on renin levels, without a thorough investigation of aldosterone or ARR changes [18–20]. There is some evidence that doxazosin may marginally increase renin release [21–23], but no notable investigations have been performed regarding the effect of doxazosin on aldosterone levels nor the ARR beyond our own and that of Mulatero and colleagues.

Unlike hydralazine and doxazosin, verapamil has had a fair amount of scientific investigation regarding its effects on renin and, to a lesser extent, aldosterone. While the vast majority of studies found that renin and aldosterone were unaffected by verapamil [24–31], this was not a universal finding. For example, Sun and colleagues noted that verapamil resulted in a significant rise in PRA in their study [32]. They hypothesized that this was the result of l-type calcium channel blockade resulting in higher intracellular calcium levels and consequently lower renin uptake in blood cells and, therefore, higher renin concentrations in plasma. Zacharieva and coworkers, as well as Frishman and colleagues, also noted a rise in PRA upon verapamil administration [33, 34]. Conversely, Buckley and coworkers found that verapamil suppressed the entire RAAS system [35]. Concerningly, there is evidence that verapamil attenuates the aldosterone response to angiotensin-II [36–40]. This could be a possible explanation for the non-significant decline noted in our own investigation. It should be stressed that while verapamil has been fairly thoroughly investigated, vis-a-vis, its effect on renin levels, the volume of research regarding aldosterone is far from comprehensive. To date, to the best of our knowledge, our study is the only investigation performed observing the effect of verapamil on individual ARR levels.

The primary clinical implication of our findings is a warning against complacency with regard to the usage of the abovementioned drugs when screening for PA. The level of research regarding these drugs and their effect on the ARR is simply far from authoritative. While it is true that these drugs have far less direct interaction with the RAAS in their primary mechanisms of action than many other antihypertensives and thus theoretically are less likely to affect the ARR values of patients undergoing PA screening, it is unwise to assume that they are inert when screening for PA. Such a lack of prudence would in all likelihood result in a significant number of missed diagnoses.

We hope that these findings demonstrate the clear and pressing need for greater research on this topic. Almost no research regarding these drugs and their effect on the ARR have been performed, and inasmuch as data are available, the majority of these are now roughly 40 years old.

The main weakness of our study was that our patients had blood samples taken from the supine position, despite current guidelines advising that patients be in the seated position for blood samples when screening for PA. However, we deliberately wanted to avoid posture-related variations in renin and aldosterone levels as the upright position may have variable effects on plasma aldosterone. Previous research has indicated that the ARR is less variable when measured in the supine position [41]. An additional limitation was our somewhat small population sizes. Having larger sample sizes would have given greater strength to our findings and, in the case of verapamil, would rule out the possibility of a type II error.

## Conclusions and Perspectives

Our data show that hydralazine resulted in a borderline significant rise in active plasma renin concentration and a significant fall in the aldosterone-to-renin ratio. Doxazosin caused declines in both plasma aldosterone concentration and the aldosterone-to-renin ratio. Verapamil did not significantly affect any of our outcome variables though it did result in a large but non-significant decline in both PAC and ARR. Based on our findings, we conclude that using these drugs when screening for PA with no regard for their potential effects on the ARR to be unadvisable. While it is possible that they are indeed the best option when full antihypertensive drug cessation is infeasible, the potential effects of these agents must still be taken into account when interpreting the aldosterone-to-renin ratio. Additionally, we see a clear and pressing need for further studies on this topic in larger patient groups with or without evidence of primary aldosteronism. We believe that our findings emphasize the importance of standardized test conditions when measuring the ARR in order to make accurate interpretation feasible. Clinicians should weigh up the risks and benefits of drug cessation when performing ARR testing, and when cessation isn’t viable, clinicians should be aware of the potentially unpredictable changes to the ARR that can result even by drugs deemed relatively inconsequential to the ARR. In patients using medication at the time of ARR testing, the increased likelihood of both type I and II errors should be kept in mind.

## Data Availability

All data and materials as well as software application support our claims and comply with field standards. Data are available upon request.
